# Long-Term Cancer Risk of US Service Members Exposed to Burn Pits in Iraq

**DOI:** 10.1001/jamanetworkopen.2025.4436

**Published:** 2025-04-10

**Authors:** Ian Robertson, Robert Sgrignoli, Alexander Dew, Sarah Darmon, Ola Landgren, Dickran Kazandjian, Christin DeStefano

**Affiliations:** 1 Walter Reed National Military Medical Center, Bethesda, Maryland; 2 Henry M. Jackson Foundation for the Advancement of Military Medicine, Bethesda, Maryland; 3 Murtha Cancer Center Research Programs, Bethesda, Maryland; 4 Sylvester Comprehensive Cancer Center, Miami, Florida

## Abstract

This cohort study of active-duty service members in the US military examines the association of exposure to burn pits during the war in Iraq with incidence of cancer.

## Introduction

Active-duty service members (ADSMs) who deployed to Iraq and Afghanistan were potentially exposed to burn pits, which emit carcinogenic byproducts.^1,2^ We hypothesized that burn pit exposure increases cancer risk later in life.

## Methods

A retrospective cohort study was performed to assess long-term cancer risk in ADSMs exposed to burn pits in Iraq. Inclusion criteria were ADSMs who: deployed to Joint Base Balad or Camp Taji between January 1, 2005, and June 30, 2007, for 180 days or more; reported burn pit exposure on their postdeployment health assessment forms; were aged 35 years or older during their deployment; and remained in the military 10 years or more postdeployment. To minimize confounding from overseas service and the healthy deployer effect, 2 control cohorts were included: ADSMs who served during the same time as the deployed cohort and who served overseas in Germany (control cohort 1) or never deployed (control cohort 2). Controls were individually matched to the deployed cohort by age, sex, branch of service, rank, and occupation.

Given the young age of the cohorts, incident cancers were expected in 5% of control cohorts after 10-year follow up. Approximately 450 ADSMs were needed to detect a 5% difference in incident cancers between the deployed and 2 control cohorts. Consequently, 534 ADSMs were included in both the deployed cohort and control cohort 1, while control cohort 2 comprised 521 ADSMs.

Incident cancers were captured via *International Classification of Diseases, Ninth Revision (ICD-9)* and *International Statistical Classification of Diseases and Related Health Problems, Tenth Revision (ICD-10)* codes. A Fisher test compared postdeployment malignant neoplasms between deployed ADSMs exposed to burn pits and control cohorts. Logistic regression estimated odds ratios (ORs) and 95% CIs for incident cancers. A false discovery rate (FDR) adjusted *P* value was applied to minimize the risk of type 1 error from multiple comparisons.

All data were provided by the Armed Forces Health Surveillance Division in May 2024, and all analyses were completed in July 2024. The study was exempt from full review and the requirement for informed consent by the Uniformed Services University institutional review board as it was less than minimal risk. This study followed Strengthening the Reporting of Observational Studies in Epidemiology (STROBE) reporting guideline.

## Results

Of the 1589 ADSMs included in the overall study group (median age, 37 years; range, 31-55 years), 1415 (89.0%) were male; 298 (18.8%) self-reported as Black, 118 (7.4%) as Hispanic, 1033 (65.0%) as White, and 140 (8.8%) as other race or multiple races (Table 1). Median (SD) follow-up time for the deployed cohort and 2 control cohorts was 14 (2) years.

**Table 1.** Baseline Demographics

Characteristics	Service members, No. (%)			
	Total (N = 1589)	Deployed (n = 534)	Control group 1 (n = 534)	Control group 2 (n = 521)
Age, median (range), y	37 (31-55)	37 (35-52)	37 (35-55)	37 (31-54)
Deployment duration, median, d	NA	243	NA	NA
Race				
Black	298 (18.8)	100 (18.7)	100 (18.7)	98 (18.8)
Hispanic	118 (7.4)	40 (7.5)	40 (7.5)	38 (7.3)
White	1033 (65.0)	346 (64.8)	346 (64.8)	341 (65.5)
Other^a^	140 (8.8)	48 (9.0)	48 (9.0)	44 (8.4)
Sex				
Male	1415 (89.0)	475 (89.0)	475 (89.0)	465 (89.3)
Female	174 (11.0)	59 (11.0)	59 (11.0)	56 (10.7)
Occupation				
Repair or engineering	434 (27.3)	147 (27.5)	147 (27.5)	140 (26.9)
Communication or intelligence	355 (22.3)	119 (22.3)	119 (22.3)	117 (22.5)
Infantry, artillery, or combat engineering	246 (15.5)	82 (15.4)	82 (15.4)	82 (15.7)
Health care	156 (9.8)	52 (9.7)	52 (9.7)	52 (10)
Pilot or aircrew	72 (4.5)	24 (4.5)	24 (4.5)	24 (4.6)
Motor transport	67 (4.2)	23 (4.3)	23 (4.3)	21 (4)
Other	259 (16.3)	87 (16.3)	87 (16.3)	85 (16.3)
Rank				
Enlisted	995 (62.6)	335 (62.7)	335 (62.7)	325 (62.4)
Officer	594 (37.4)	199 (37.3)	199 (37.3)	196 (37.6)
US military branch				
Army	1513 (95.2)	508 (95.1)	508 (95.1)	497 (95.4)
Air Force	76 (4.8)	26 (4.9)	26 (4.9)	24 (4.6)

Abbreviation: NA, not applicable.

^a^ The Armed Forces Health Surveillance Division data do not specify groups included in other.

The most common incident cancers diagnosed in the entire cohort were basal cell carcinoma (BCC) (43 cases), male reproductive tumors (21 cases), and colorectal cancer (12 cases) (Table 2). Incident cancers occurred in 39 service members (7.3%) in both the deployed cohort and the control cohort 1 (OR, 1.0; 95% CI, 0.63-1.59; *P* > .99), and in 60 service members (11.5%) of control cohort 2 (OR, 1.65; 95% CI, 1.08-2.52; *P* = .02, FDR-adjusted *P* = .37). Compared with the deployed cohort, control cohort 2 had a higher incidence of BCC, 4.2% (22 of 521 service members) vs 1.9% (10 of 534 service members) (OR, 2.31; 95% CI, 1.08-4.93; *P* = .03, FDR-adjusted *P* = .37), and male reproductive tumors, 2.3% (12 of 521 service members) vs 0.7% (4 of 534 service members) (OR, 3.12; 95% CI, 1.00-9.75; *P* = .045, FDR-adjusted *P* = .37); these findings were not statistically significant after FDR correction. There were no differences in other incident cancers between the deployed and 2 control cohorts.

**Table 2.** Incident Cancers Diagnosed After Long-Term Follow Up

Cancer type	No. total	Deployed		Control 1		Control 2		*P* value					
		Cases, No. (%)	OR (95% CI)	Cases, No. (%)	OR (95% CI)	Cases, No. (%)	OR (95% CI)	Overall	Control 1 vs deployed	Control 2 vs deployed	FDR-adjusted		
											Overall	Control 1 vs deployed	Control 2 vs deployed
All	138	39 (7.3)	1 [Reference]	39 (7.3)	1.00 (0.63-1.59)	60 (11.5)	1.65 (1.08-2.52)	.02	>.99	.02	.37	>.99	.37
Skin	69	18 (3.4)	1 [Reference]	19 (3.6)	1.06 (0.55-2.04)	32 (6.1)	1.88 (1.04-3.39)	.06	>.99	.04	.37	>.99	.37
BCC	43	10 (1.9)	1 [Reference]	11 (2.1)	1.10 (0.46-2.62)	22 (4.2)	2.31 (1.08-4.93)	.044	>.99	.03	.37	>.99	.37
SCC	5	2 (0.4)	1 [Reference]	1 (0.2)	0.50 (0.05-5.52)	2 (0.4)	1.03 (0.14-7.30)	.87	>.99	>.99	>.99	>.99	>.99
Melanoma	3	1 (0.2)	1 [Reference]	2 (0.4)	2.00 (0.18-22.16)	0	NA	.78	>.99	>.99	>.99	>.99	>.99
Other	18	5 (0.9)	1 [Reference]	5 (0.9)	1.00 (0.29-3.47)	8 (1.5)	1.65 (0.54-5.08)	.64	>.99	.42	>.99	>.99	.92
Reproductive	24	7 (1.3)	1 [Reference]	5 (0.9)	0.71 (0.22-2.26)	12 (2.3)	1.78 (0.69-4.54)	.20	.77	.25	.68	>.99	.73
Male	21	4 (0.7)	1 [Reference]	5 (0.9)	1.25 (0.33-4.69)	12 (2.3)	3.12 (1.00-9.75)	.07	>.990	.045	.39	>.99	.37
Female	3	3 (0.6)	1 [Reference]	0	NA	0	NA	.11	.25	.25	.44	.73	.73
Colorectal	12	2 (0.4)	1 [Reference]	6 (1.1)	3.02 (0.61-15.04)	4 (0.8)	2.06 (0.38-11.28)	.37	.29	.45	.90	.79	.95
Thyroid	6	4 (0.7)	1 [Reference]	1 (0.2)	0.25 (0.03-2.23)	1 (0.2)	0.26 (0.03-2.29)	.38	.37	.37	.90	.90	.90
Blood	5	2 (0.4)	1 [Reference]	0	NA	3 (0.6)	1.54 (0.26-9.26)	.22	.50	.68	.71	.97	>.99
Breast	5	2 (0.4)	1 [Reference]	1 (0.2)	0.50 (0.05-5.52)	2 (0.4)	1.03 (0.14-7.30)	.87	>.99	>.99	>.99	>.99	>.99
Kidney	5	1 (0.2)	1 [Reference]	3 (0.6)	3.01 (0.31-29.05)	1 (0.2)	1.03 (0.06-16.43)	.63	.62	>.99	>.99	>.99	>.99
Brain	3	2 (0.4)	1 [Reference]	1 (0.2)	0.50 (0.05-5.52)	0	NA	.78	>.99	.50	>.99	>.99	.97
Sarcoma	3	0	1 [Reference]	0	NA	3 (0.6)	NA	.04	NA	.12	.37	NA	.44
Other	6	1 (0.2)	1 [Reference]	3 (0.6)	3.01 (0.31-29.03)	2 (0.4)	2.05 (0.19-22.71)	.71	.62	.62	>.99	>.99	>.99

Abbreviations: BCC, basal cell carcinoma; NA, not applicable; SCC, squamous cell carcinoma.

## Discussion

Our study did not demonstrate an increased risk of cancer among ADSMs who served in Iraq and were exposed to pits. However, there were more incident cancers among never deployed, namely BCC and male reproductive tumors, although these findings did not reach statistical significance after FDR-adjustment. Limitations of this study are potential confounding from recall bias, heterogeneous individual exposure levels, and inability to capture health care data from the Veterans Health Administration (VHA) or civilian hospitals on ADSMs after they leave the military.^3^ Stronger collaborations between the Department of Defense, VHA, and civilian cancer registries could help capture more longitudinal health care data on a larger number of military members postservice to better characterize long-term cancer risk.^4^

## Notes

Supplement.
Data Sharing Statement
